# Use of mepolizumab in adult patients with cystic fibrosis and an eosinophilic phenotype: case series

**DOI:** 10.1186/s13223-019-0397-3

**Published:** 2020-01-06

**Authors:** Lijia Zhang, Larry Borish, Anna Smith, Lindsay Somerville, Dana Albon

**Affiliations:** 1 University of Virginia School of Medicine, Charlottesville, VA 22908 USA; 2 Department of Medicine, University of Virginia School of Medicine, PO Box 800546, Charlottesville, VA 22908 USA; 3 Department of Microbiology, University of Virginia School of Medicine, Charlottesville, VA 22908 USA

**Keywords:** Cystic fibrosis, Mepolizumab, Asthma, Allergic bronchopulmonary aspergillosis/mycosis, Type 2 inflammation

## Abstract

**Background:**

Cystic fibrosis (CF) is characterized by inflammation, progressive lung disease, and respiratory failure. Although the relationship is not well understood, patients with CF are thought to have a higher prevalence of asthma than the general population. CF Foundation (CFF) annual registry data in 2017 reported a prevalence of asthma in CF of 32%. It is difficult to differentiate asthma from CF given similarities in symptoms and reversible obstructive lung function in both diseases. However, a specific asthma phenotype (type 2 inflammatory signature), is often identified in CF patients and this would suggest potential responsiveness to biologics targeting this asthma phenotype. A type 2 inflammatory condition is defined by the presence of an interleukin (IL)-4^high^, IL-5^high^, IL-13^high^ state and is suggested by the presence of an elevated total IgE, specific IgE sensitization, or an elevated absolute eosinophil count (AEC). In this manuscript we report the effects of using mepolizumab in patients with CF and type 2 inflammation.

**Results:**

We present three patients with CF (63, 34 and 24 year of age) and personal history of asthma, who displayed significant eosinophilic inflammation and high total serum IgE concentrations (type 2 inflammation) who were treated with mepolizumab. All three patients were colonized with multiple organisms including *Pseudomonas aeruginosa* and *Aspergillus fumigatus* and tested positive for specific IgE to multiple allergens. We examined the effect of mepolizumab on patients’ lung function (FEV1), blood markers of type 2 inflammation, systemic corticosteroid use and frequency of CF exacerbations. One patient had a substantial increase in lung function after starting mepolizumab and all three patients had a substantial benefit in regards to reduced oral CCS use. While none of the patients showed significant changes in the exacerbation rates there was markedly reduced requirements for oral CCS with exacerbations. In addition, mepolizumab had a positive effect on type 2 inflammatory markers, reducing markers of allergic inflammation in all 3 patients.

**Conclusions:**

Mepolizumab appears to have a positive effect on clinical course in patients with CF presenting with a type 2 phenotype characterized by allergic sensitization and hyper-eosinophilia.

## Background

Cystic fibrosis (CF) is caused by an autosomal recessive function deficiency of the transmembrane regulator (CFTR) protein [1]. CFTR is a cAMP regulated chloride channel that is expressed on the apical membranes of epithelial cells [2]. These changes can lead to significant defects in host anti-bacterial defenses. CF is characterized by extensive inflammation, progressive lung disease, and respiratory failure. Neutrophils, B and T-lymphocytes are markedly increased within the airway epithelium [3, 4]. Although the relationship is not well understood, patients with CF are thought to have a higher prevalence of asthma than the general population. CF Foundation (CFF) annual registry data in 2017 reported a prevalence of asthma in CF of 32%. It is difficult to differentiate asthma from CF given similarities in symptoms, lung function variability, and bronchodilator responses in both diseases. However, a specific asthma phenotype characterized by a type 2 inflammatory signature can often be identified in CF patients.

A type 2 inflammatory lung condition is defined by the presence of an interleukin (IL)-4^high^, IL-5^high^, IL-13^high^ state. This signature can reflect cytokine production by both the adaptive immune [type 2 T helper (Th2) effector lymphocytes] or innate immune system [innate lymphoid type 2 (ILC2) cells, mast cells, eosinophils, and others] [5, 6]. Type 2 (T2) inflammation is suggested by the presence of elevated total immunoglobulin E (IgE), specific IgE sensitization (determined via ImmunoCap^®^ assay), or elevated absolute eosinophil count (AEC) in the circulation. But more compelling evidence is the presence of increased eosinophil markers, type 2 cytokines, or type 2 cytokine-producing cells in the airway (or sputum), and possibly also elevated numbers of circulating Th2 effector lymphocytes [7–9]. In CFTR-deficient mice, naïve CD4^+^ T-cells demonstrate a Th2 bias in vitro in response to *Aspergillus fumigatus*, with elevated IL-4 in the lungs and allergen-specific IgE in the serum. Moreover, CFTR knockout T cells demonstrate a bias to develop a robust Th2 response to *Aspergillus fumigatus* antigens, with increased levels of IL-13 and IL-4 [10, 11]. This Th2 bias has also been studied in patients with *Pseudomonas aeruginosa*-infections, who have higher levels of pulmonary CCR4^+^CD4^+^ (Th2) effector cells and elevated expression of IL-4 and IL-13. These levels correlated inversely with FEV1 [12]. In the absence of *Pseudomonas*, a robust type 2 response represents an independent risk factor for future infection with this pathogen, suggesting that type 2 inflammation can be a stereotypic response that develops independently of infection [13].

Mepolizumab is a humanized monoclonal antibody of targeting IL-5 that offers therapeutic benefits for eosinophilic asthma [14]. In severe asthma with high blood eosinophil counts, it reduces exacerbation frequency by 32% or more and also has a corticosteroid-sparing effect [15]. However, there is limited research on the effects of mepolizumab in the treatment of CF patients with concomitant type 2 inflammation.

We speculated that targeting the type 2 immune response in CF patients might attenuate asthmatic inflammation and thereby improve lung function, slow remodeling, and decrease exacerbation rates, especially in patients whose exacerbations were associated with eosinophilic inflammation. In this article, we present three patients with CF and significant eosinophilic inflammation who were treated with mepolizumab. We examined the effect of mepolizumab on patients’ lung function (FEV1), blood markers of type 2 inflammation [indicated by total IgE and Absolute Eosinophil Count (AEC)], and frequency of CF exacerbations.

## Case presentation

**Fig. 1:**
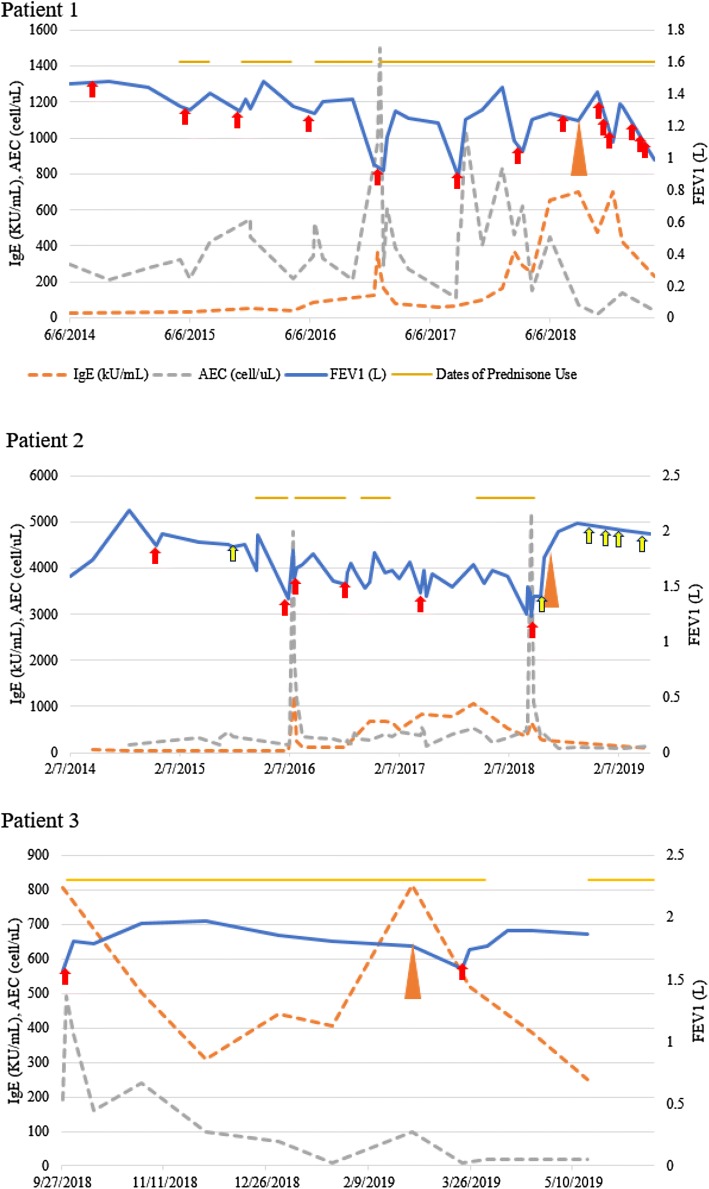
Clinical course of patients receiving mepolizumab for CF with evidence of type 2 inflammation. Red and yellow arrows represent episodes of CF exacerbations associated with lung function decline, requiring intravenous or oral antibiotics, respectively. Orange triangle represents mepolizumab initiation. The yellow lines across the top of each figure represent periods of systemic corticosteroid use

*Patient 1* is a 63-year-old white woman with CF homozygous delF508. She was colonized chronically with *Pseudomonas aeruginosa*, *methicillin resistant Staphylococcus aureus* (MRSA), *Aspergillus fumigatus*, and *Exophiala*. Over the past 5 years, she presented yearly with 3 or more CF exacerbations requiring admission to the hospital and intravenous antibiotics. Beginning in 2016, during her exacerbations, she developed positive markers for type 2 inflammation (Fig. 1a), with eosinophil levels ranging from 300 to 1500/µL and IgE levels of 25–700 IU/mL and pulmonary infiltrates on Chest X-ray (CXR). Specific IgE testing revealed positive results for cat (she had two indoor cats) and dog dander and 2 fungi (*Alternaria alternata, Aspergillus fumigatus*). A diagnosis of allergic bronchopulmonary aspergillosis (ABPA) was entertained. During exacerbations, she presented with increased cough, sputum production, chest congestion, chest tightness and wheezing that was relieved by systemic corticosteroid (CCS) treatment. Subsequently, she became CCS dependent. She also received fluticasone/salmeterol metered dose inhaler and montelukast. Ivacaftor/Tezacaftor was started in 2018 after FDA approval. In an effort to decrease systemic CCS in context of difficult to control CFRD, she was begun on therapy with mepolizumab (100 mg SQ every 28 days) on July, 2018. Prior to initiation of mepolizumab, baseline FEV1 was 60% predicted (calculated as the mean between two best values over 12 months) and she demonstrated large variability in her FEV1% predicted (variability was 23% calculated as the difference between the highest and the lowest FEV1 over 12 months). After beginning mepolizumab she was able to decrease her prednisone use from 20 to 5 mg daily. However, her baseline FEV1 percent predicted did not change and she continued to have large variability in FEV1% predicted (20%) between measurements. Of note, 9 months after starting mepolizumab her total IgE concentration normalized (to as low as 148 kU/L).

*Patient 2* is a 34-year-old white woman with CF, homozygous delF508. Over the past 5 years, she presented consistently with 1–2 CF exacerbations yearly requiring admission to the hospital and intravenous antibiotics. Her FEV1% predicted showed large variability, as high as 28% between measurements, and her baseline FEV1% predicted was 55%. Chronically, she was colonized with *Pseudomonas aeruginosa*, *Burkholderia cepacia (Multivorans), methicillin sensitive Staphylococcus aureus* (MSSA), *Aspergillus fumigatus*, and *Exophiala*. In 2016, during her exacerbations, she developed positive markers for type 2 inflammation, with AECs ranging from 200 to 1200/µL and IgE levels of 40–1250 IU/mL (Fig. 1b). Specific IgE testing revealed specific IgE for *Alternaria alternata*. During exacerbations, she presented with increased cough, sputum production, chest congestion, pulmonary infiltrates on chest imaging and also chest tightness and wheezing that was relieved by systemic CCS treatment. Her routine maintenance CF therapy included: nebulized albuterol, hypertonic saline and dornase alpha twice daily, azithromycin (Monday, Wednesday and Friday). She used inhaled aztreonam and tobramycin in alternating months. She also received budesonide/formoterol inhaled and montelukast. The elevated eosinophils and IgE in the setting of a high specific IgE to *Alternaria*, were concerning for allergic bronchopulmonary mycosis (ABPM) and she was advised to start oral prednisone. Given hard to control CFRD, the patient requested alternative therapy to prednisone. In an effort to decrease systemic CCS use, she was recommended to be begun on mepolizumab and she initiated this therapy in June, 2018. Ivacaftor/tezacaftor was started in 2018 and initiation of this therapy coincided with that of mepolizumab. After beginning mepolizumab (and tezacaftor/ivacaftor), her FEV1% predicted increased from a baseline of 55% to 70% and her IgE normalized (Fig. 1b) and the variability between FEV1 measurements decreased from 28% predicted to 3%. After starting mepolizumab, she did develop 4 mild exacerbations requiring oral antibiotics, however, her lung function remained stable during these exacerbations. Prior to mepolizumab, she received short courses of prednisone with each exacerbation, which is no longer the case. She remains oral CCS free and her CFRD is well controlled with Hba1c of 6.0%.

*Patient 3* is a 24-year-old white woman with CF, heterozygous delF508/1461INF4. Chronically, she was colonized with *Pseudomonas aeruginosa*, *methicillin susceptible Staphylococcus aureus* (MSSA), and *Aspergillus fumigatus*. In September, 2018, she developed an acute CF exacerbation associated with positive markers for type 2 inflammation, with IgE levels of 800 IU/mL and an AEC of 490/µL (Fig. 1c). Specific IgE testing was positive for cat and dog dander and 3 fungi (*Alternaria alternata, Aspergillus fumigatus,* and *Cladosporium herbarum*). A diagnosis of ABPA was made and she initiated prednisone and antifungal therapy. However subsequently she became CCS-dependent and multiple attempts to taper the prednisone led to CF/ABPA exacerbations. She also received budesonide/formoterol inhaled and montelukast in addition to routine CF care. In an effort to decrease systemic CCS, especially in the context of difficult to control CFRD, she was initiated in February, 2019. Prior to initiation, she had had a baseline FEV1% predicted of 60%. After beginning mepolizumab, her prednisone was successfully tapered from 20 to 5 mg daily and her CFRD has been better controlled with Hba1c improving from 10.8 to 7.0%. In addition, she developed 2 exacerbations secondary to respiratory viral infections requiring IV antibiotics, however, her lung function remained stable. Her FEV1 has not changed. Finally, since starting mepolizumab, her total IgE has decreased to 288 IU/mL.

## Discussion and conclusion

We present three patients who received mepolizumab for type 2 inflammation. All three patients were colonized with *Pseudomonas aeruginosa* and *Aspergillus fumigatus* (and/or other fungi) and had positive specific fungal IgE. As such, the diagnosis of ABPA/ABPM was entertained in all 3 patients. It is important to appreciate, however, that it is difficult (or perhaps even impossible) to distinguish ABPA from type 2 high fungal allergen-exacerbated asthma in the CF population as many of the features that distinguish ABPA from allergic asthma in the non-CF population (presence of bronchiectasis, fleeting infiltrates, bronchial casts) are characteristics of CF and that both type 2 high fungal allergen-exacerbated asthma and ABPA/ABPM demonstrate elevated total and specific IgE and eosinophilia). All these patients were treated with continuous nebulized antibiotics; they received inhaled corticosteroids/long-acting beta agonists and montelukast as well as CCS without improvement in their symptoms or in type 2 inflammatory markers. They required either multiple bursts of prednisone (patient 2) or were prednisone dependent (patients 1 and 3). Patients 1 and 2 received tezacaftor/ivacaftor (which was started at the same time as the mepolizumab in patient 2 making it difficult to categorically ascribe the clinical benefit to the biologic).

Mepolizumab was well tolerated. Patient 2 had a substantial increase in FEV1 after starting mepolizumab. The FEV1% predicted increased from 55 to 70% predicted and stabilized and there was significantly less variability between measurements. She developed no exacerbations requiring IV antibiotics. In the 6–12 months follow-up post-mepolizumab, none of the patients showed significant changes in the exacerbation rates. None of the patients had a baseline FEV1 decline post initiation of mepolizumab. Most importantly, all three patients had a substantial benefit in regards to oral CCS use: Patient 2 required no corticosteroid bursts after starting mepolizumab and patients 1 and 3 were able to decrease the prednisone use to 5 mg daily. All three patients had a positive response to mepolizumab in regards to IgE levels. After starting mepolizumab, patients 2 and 3 had an immediate decline in both eosinophil and IgE levels. For patient 1 it took almost a year of this anti-eosinophil therapy to see normalization of IgE. We suspect this could be due to persistent allergen exposure, as she slept in the same bedroom with her two cats.

We conclude that mepolizumab has a positive effect on type 2 inflammation in patients with CF presenting with an eosinophilic phenotype. Mepolizumab is safe and easily tolerated in patients with CF and type 2 inflammation. The limitations of our study are related to a low number of subjects, absence of a control cohort, and retrospective data collection. The diagnosis of ABPA/M was at least entertained in all 3 subjects so our results might not extend to all CF patients with type 2 inflammation. Further studies are needed to demonstrate a positive effect of anti-eosinophilic treatment in patients with CF and eosinophilic, type 2 inflammation.

## Data Availability

The datasets used and/or analyzed during the current study are available from the corresponding author on reasonable request.

## Abbreviations

ABPA: allergic bronchopulmonary aspergillosis
ABPM: allergic bronchopulmonary mycosis
AEC: absolute eosinophil count
CF: cystic fibrosis
CFF: Cystic Fibrosis Foundation
CFRD: CF-related diabetes
CFTR: CF transmembrane regulator
CCS: corticosteroids
FEV1: forced expiratory volume in 1 s
Ig: immunoglobulin
IL: interleukin
ILC: innate lymphoid cell
MRSA: methicillin resistant *Staphylococcus aureus*
MSSA: methicillin sensitive *Staphylococcus aureus*
Th2: T helper 2
